# An Exploration of Features Impacting Respiratory Diseases in Urban Areas

**DOI:** 10.3390/ijerph19053095

**Published:** 2022-03-06

**Authors:** Ihsane Gryech, Mounir Ghogho, Chafiq Mahraoui, Abdellatif Kobbane

**Affiliations:** 1 TICLab Research Laboratory, International University of Rabat, Rabat 11103, Morocco; mounir.ghogho@uir.ac.ma; 2 ENSIAS, Mohammed V University in Rabat, Rabat 10100, Morocco; abdellatif.kobbane@um5.ac.ma; 3 School of Electronic and Electrical Engineering, The University of Leeds, Leeds LS2 9JT, UK; 4 Centre Hospitalo-Universitaire Ibn Sina de Rabat—CHUIS, Rabat 10100, Morocco; cmahraoui@gmail.com

**Keywords:** air quality, respiratory health, features abstraction, geographical information systems, open data set, random forest

## Abstract

Air pollution exposure has become ubiquitous and is increasingly detrimental to human health. Small Particulate matter (PM) is one of the most harmful forms of air pollution. It can easily infiltrate the lungs and trigger several respiratory diseases, especially in vulnerable populations such as children and elderly people. In this work, we start by leveraging a retrospective study of 416 children suffering from respiratory diseases. The study revealed that asthma prevalence was the most common among several respiratory diseases, and that most patients suffering from those diseases live in areas of high traffic, noise, and greenness. This paved the way to the construction of the MOREAIR dataset by combining feature abstraction and micro-level scale data collection. Unlike existing data sets, MOREAIR is rich in context-specific components, as it includes 52 temporal or geographical features, in addition to air-quality measurements. The use of Random Forest uncovered the most important features for the understanding of air-quality distribution in Moroccan urban areas. By linking the medical data and the MOREAIR dataset, we observed that the patients included in the medical study come mostly from neighborhoods that are characterized by either high average or high variations of pollution levels.

## 1. Introduction

**Figure 1:**
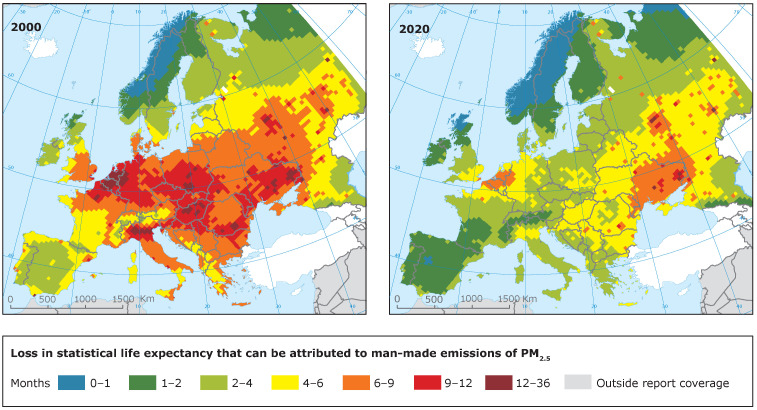
Increase of life expectancy due to reduction of ${\mathrm{PM}}_{2.5}$ emissions from year 2000 (**left**) to year 2020 (**right**) [2]. Copyright holder: European Environment Agency (EEA).

Air pollution is the largest environmental risk to health. According to the World Health Organization, around 6.5 million deaths per year are the result of air pollution-related conditions. Of those, 4.2 million are attributable solely to outdoor air pollution [1]. Due to the devastating burden resulting from air pollution, significant efforts have been placed on the investigation and regulation of air pollution. In Europe, for instance, even though pollution remains an important risk factor, it has decreased remarkably over the last decade, which has led to a visible reduction in loss of life expectancy due to exposure to ${\mathrm{PM}}_{2.5}$ (Figure 1).

Geographically, mortality from air pollution is dominated by East Asia (35%) and South Asia (32%), followed by Africa (11%) and Europe (9%) [3]. Aside from mortality, short- and long-term exposure to high levels of pollution can impact the respiratory, cardiovascular, ophthalmologic, dermatologic, neuropsychiatric, hematologic, immunologic, and reproductive systems [4,5]. The respiratory system is the first to be damaged since pollutants, especially particulate matter, enter the body through the airways [6,7,8]. Exposure studies using concentrated air particulates have demonstrated that ambient air particulates cause airway inflammation [9,10] and that pollution sources are directly linked to asthma exacerbation [11].

More than 13,000 deaths are caused by air pollution in Morocco each year, representing about 7% of all deaths, thus making it the 8th highest mortality risk factor [12]. During the COVID-19 lockdown period in Morocco, an examination of the number of deaths related to PM exposure was conducted, revealing a marked decrease in both ambient air PM levels and PM-related deaths [13].

Outdoor air pollution originates from both natural and anthropogenic sources. Natural sources contribute to local air pollution in rural regions and in regions prone to forest fires and dust storms. However, man-made sources still exceed natural ones and have a higher impact on air pollution. The best-known air pollution sources defined by the World Health Organization (WHO) are: fuel combustion from motor vehicles, heat and power generation, industrial facilities, municipal and agricultural waste sites and waste incineration/burning, as well as residential cooking, heating, and lighting with polluting fuels.

Pollution sources can differ from one region to another as well as from one continent to another. In Africa, specifically in the case of Morocco, residential heating and lighting with fuels are not common and thus do not contribute to poor air quality. However, food-processing and waste-burning are more usual practices and do indeed contribute to poor air quality [14].

Several countries have begun providing access to outdoor air-quality data collected by monitoring agencies such as “Air Data” in the United States [15] and “Airparif” in France [16]. Although these datasets provide hourly measurements of air quality, they do not specify what produces that pollution, or the different factors responsible for it. In addition, even though such datasets can be incredibly useful to the monitoring of air pollution, they do not provide much insight into pollution sources or on their impact.

In the case of Morocco, there are no publicly available datasets of continuous measurements of air quality, nor is there a resource highlighting the different pollution sources and impacting factors.

In a previous work [17], we assessed the status of asthma in Morocco by investigating perinatal, prenatal, postnatal and indoor environmental risk factors. In this work, we aim to tackle outdoor environmental factors and study the impact of pollution on children’s health. To that end, we adopt a bottom-up approach by first selecting patients suffering from respiratory diseases and investigating potential impacting factors. The main contributions of this work are:

- We conduct a medical investigation of respiratory disease patients using data from hospitalized children in Rabat, Morocco.
- We investigate the surroundings and environment of these patients and compose a list of potential pollution sources.
- We propose a context-specific dataset, coined MOREAIR, using a novel data collection methodology.
- We use machine learning to determine the factors that most impact air quality and pollutant distribution within the Moroccan context.
- We investigate how air quality in Morocco varies over time and space.

## 2. Medical Investigation

Through our collaboration with the Ibn Sina Hospital Center (CHUIS), we were provided with access to data gathered from the children’s hospital registers in Rabat, Morocco. It is worth noting that CHUIS is the only public hospital center specializing in respiratory diseases in the city of Rabat and its outskirts.

### 2.1. Medical Records Description

A retrospective study of 416 children was previously conducted in the pneumology, allergology and infectiology service at the Ibn Sina’s Children Hospital Center (CHUIS). A dataset resulting from this study was made available to us. The study consists of children who were hospitalized for a moderate to severe respiratory disease. The data collection was conducted over a period of 4 months, from January to April 2018. The age of the children included in the study varies from 11 days to 14 years. The doctors participating in the study interviewed the child, as well as the child’s mother in their native language (Moroccan dialect). The questionnaire surveys used for the interviews were designed and implemented by pediatricians to identify significant factors that are potentially associated with childhood asthma. In this questionnaire, patients’ files reported the following events:

- Name, gender, weight, admission date, address, and social status.
- Information about their households and their surroundings: presence of green spaces nearby, busy roads, junctions, exposure to outdoor dust, smoke and noise.
- Description of their medical history, genetic disorders, duration of breastfeeding, type of childbirth delivery and many other specific aspects.
- Additional information provided by patients and/or parents as well as any remarks or concerns about what impacts their health and what could be responsible for the aggravation of their respiratory discomfort.

To the best of our knowledge, the legal guardians’ verbal consent has been obtained by the pediatricians conducting the interview. From these data, we selected relevant information related to outdoor pollution. The rest of the data are out of the scope of this paper and have already been addressed in our previous study.

### 2.2. Medical Data Set Analysis

**Figure 2:**
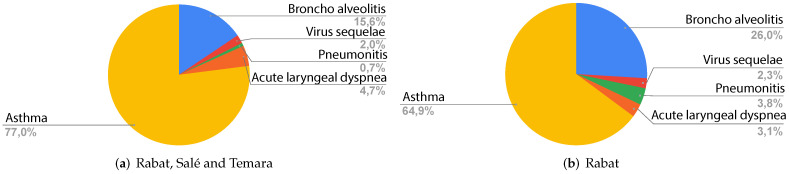
Diagnosis of admitted patient for different regions.

Figure 2 shows the different respiratory problems in the children admitted to the emergency department. For Rabat, Salé and Temara (Figure 2a, asthma is the most common diagnosis with a percentage of 77.0%, followed by bronchoalveolitis with 15.6%. Acute laryngeal dyspnea, virus sequelae and pneumonitis are less common than the aforementioned diagnoses with percentages of 4.7%, 2.0% and 0.7% respectively.

For Rabat, Figure 2b shows that asthma is the most common disease with 64.9%, followed by bronchoalveolitis with 26.0%, pneumonitis with 3.8%, acute laryngeal with 3.1% and finally virus sequelae with 2.3%.

Table 1 presents a description of the diseases mentioned in Figure 2. All these diseases are either directly or indirectly associated with air pollution and are known for their aggravated respiratory symptoms, especially in susceptible populations such as children and elderly people [18].

Based on the geographical location of the patients, as well as pediatricians’ reported information on air pollution in the patients’ surroundings, we were able to detect three major air pollution axes of potential impacting factors causing, or aggravating the patients’ respiratory state: living near green spaces, living near busy roads, and living in a noisy neighborhood. We start from the hypothesis that these three axes may have direct toxic effects on the patients’ respiratory state.

**Figure 3:**
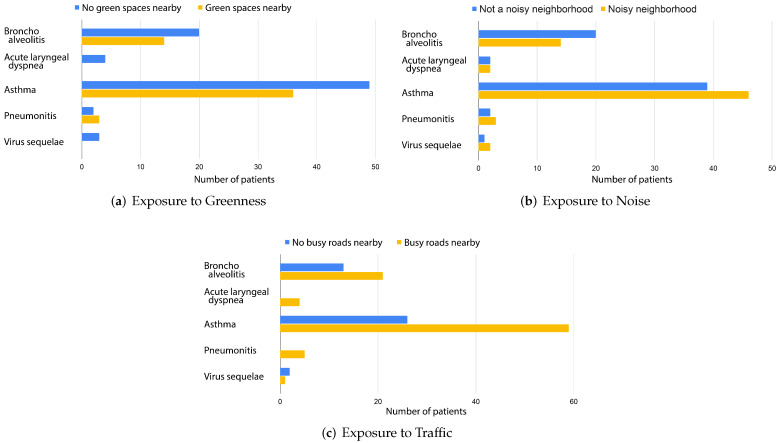
Patients’ exposure to potential source of pollution near their households.

Figure 3a depicts the distribution of patients in Rabat living near green spaces and their diagnosis. Of the 131 children admitted to the emergency department during the period covered by the registry in Rabat, 76 patients (approximately 58%) do not live near green spaces. These patients presented with bronchoalveolitis (23.68%), acute laryngeal dyspnea (7.89%), asthma (61.84%), pneumonitis (2.63%) and virus sequelae (3.94%).

These findings align with results from the literature specifying that children with respiratory diseases living near greenness such as trees, plants, and shrubs suffer from fewer respiratory symptoms [28,29]. According to the European Lung Foundation, adults who lived near green spaces during childhood suffer from fewer respiratory problems such as asthma and wheezing [30]. Thus, the 42% patients (Figure 3a) who live near green spaces could have better chances of not worsening their respiratory condition as adults.

Chronic noise exposure is known to act as an unspecific stressor. In [31], it was shown that noise annoyance is associated with increased asthma prevalence. We make the conjecture that living in a noisy neighborhood is a potentially important parameter in Morocco. A noisy neighborhood can indicate the presence of several activities causing pollution. In some neighborhoods, we can find traditional markets, street food vendors, construction sites, mechanical shops, blacksmith shops and many other activities that could be considered to be a main man-made source of pollution. Figure 3b reveals that most asthma, pneumonitis and virus sequelae patients live in a noisy neighborhood with percentages of 54%, 60% and 67% respectively. On the other hand, 41% and 50% of bronchoalveolitis and acute laryngeal dyspnea patients do not live in a noisy neighborhood. Hence, we highlight the necessity of understanding and characterizing the different sources engendering noise.

Busy roads are where several pollutants are condensed such as carbon monoxide (CO), oxides of nitrogen (NOx), hydrocarbon (HC), lead, and particulate matter [32]. Figure 3c depicts the distribution of patients who are exposed to traffic. It is shown that approximately 69% of the assessed children for all diagnoses live near road traffic. These patients presented with bronchoalveolitis (23.33%), acute laryngeal dyspnea (6.66%), asthma (63.33%), pneumonitis (5.55%), and virus sequelae (1.11%).

These findings shed some light on the potential link between the onset or aggravation of respiratory diseases and the surrounding environment of patients. Additionally, these results offer some insight into the nature of factors that could impact the respiratory state of patients in Morocco. For instance, busy roads seem to be more strongly linked to pneumonitis and acute laryngeal dyspnea cases than to asthma. This could be explained by the fact that asthma is multi-factorial and can be triggered by allergens, viruses and the internal environment while pneumonitis and acute laryngeal dyspnea are mainly caused by viruses and infections.

Although this data exploration allows for some insight into these patient environments, it also leads to several questions:

- Are common features such as traffic and green spaces the most impacting factors on pollution and human respiratory health in urban areas?
- What is noise pollution related to and what are the polluting factors found in noisy neighborhoods in Morocco?
- What is the impact of context-specific features on air quality, and how does that affect respiratory diseases?

To answer these interrogations, we consider exposure to greenness, exposure to noise, and exposure to traffic as a starting point to investigate the most context-specific features correlated with air pollution in Morocco. To contribute to the assessment of air quality in Morocco, these context-specific features will be assembled in a dataset for the region of Rabat-Salé-Temara.

## 3. MOREAIR Data Set

### 3.1. Objective and Data Collection Design

Surveys in healthcare are an important assessment tool to improve physician knowledge and remain a frequently used research study design in the medical field. Moreover, surveys can even serve as a starting point, a motivation to dive into the different areas related to that initial subject. Commonly perceived asthma factors include family history, childhood viral infections, early allergen exposure, and air pollution [33].

In this work, we mainly focus on outdoor air pollution. We use different approaches to form a comprehensive dataset covering air-quality data, temporal data related to road traffic and meteorology, as well as spatial data related to the changes encountered from one area to another.

To construct our dataset, we use a bottom-up approach by first selecting both respiratory disease patients and healthy volunteers distributed over the city of Rabat and its outskirts. We use the volunteers’ households as the center of our research area, and cover the surface of the circle surrounding it with a radius of 1000 m. Using volunteers, we cover the whole city of Rabat. The previously conducted respiratory state investigation, the geographical location of patients, as well as the pediatrician’s reported information on air pollution in the patients’ surroundings, motivated us to detect three major air pollution axes (mentioned in Section 2.2) as our starting point, and we search for features related to each axis:

- Greenness: Natural pollutants.
- Noise: Outdoor commercial activities and types of buildings.
- Traffic-related features: Transportation and types of roads.

We use geographic abstraction as well as a micro-level scale data collection to find the features found in each area and the elements that could potentially impact the health of citizens.

### 3.2. Participants

**Figure 4:**
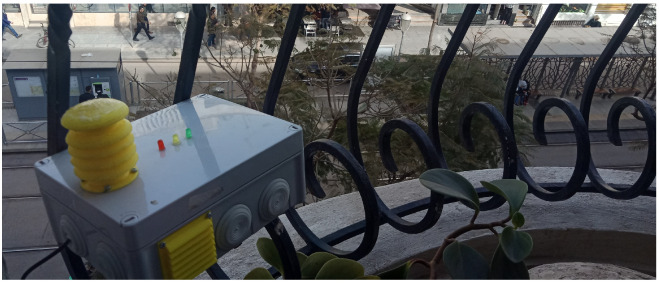
Nomadic sensor node installation.

Several of the patients mentioned earlier volunteered to install air-quality sensors in their households and allow us to measure air quality near their homes. We selected eight volunteers who suffer from asthma or a respiratory disease and eight who had no respiratory problems. These volunteers cover a total of 12 neighborhoods in Rabat, Morocco. No medical criteria were included in our selection for patients. Our main selection criterion was the geographical distribution. An important aspect of our choice also involved including both affluent and disadvantaged neighborhoods. We installed our air-quality sensor nodes in the windows of volunteers’ houses, as can be seen in Figure 4. It is worth noting that verbal consent of all participating residents was gained prior to any data acquisition. In addition, anonymity was conserved in all gathered data to maintain the volunteers’ right to privacy.

### 3.3. Temporal Data

#### 3.3.1. Air-Quality Data

Air-quality data are collected using two categories of low-cost sensors developed in our laboratories:

- MOREAIR AQ-N: Nomadic sensor nodes with a better power supply but settled in one specific location during the collection process.
- MOREAIR AQ-M: Mobile sensor nodes that operate on high-capacity batteries but have a lower energy autonomy (around 24 h). The mobile sensor nodes are used to collect data while on the move to capture local concentration variability and detect hot-spots.

Among the several components included in the sensor node [34], the concentrations of ${\mathrm{PM}}_{10}$ and ${\mathrm{PM}}_{2.5}$ are sampled using the NOVA PM SDS011 sensor, which is a digital sensor based on a laser scattering principle for a reliable, accurate and stable output quality. Light scattering can be induced when particles go through the detecting area. The scattered light is transformed into electrical signals and these signals are amplified and processed. The number and diameter of particles can be obtained by analysis because the signal waveform has certain relations with the particle’s diameter.

Air-quality data are measured using both fixed and mobile sensor nodes, which report real-time concentrations of the different air pollutants at an interval of five seconds.

Although nomadic sensor nodes are installed in patients’ households, mobile sensor nodes are carried throughout the city to collect air quality during the feature collection campaigns Section 3.4.3.

The data set constructed from the collected measurements consists of:

- ${\mathrm{PM}}_{10}$ and ${\mathrm{PM}}_{2.5}$ concentration records in $\mu {g/m}^{3}$,
- Temperature and relative humidity in °C and % respectively,
- GPS data (only for mobile sensor nodes), namely latitude, longitude and altitude,
- Timestamp associated with each measurement.

The data collection process is still ongoing at the time of writing this paper. Additional details related to each sensor node employed in the air-quality data acquisition can be found in Table 2.

**Figure 5:**
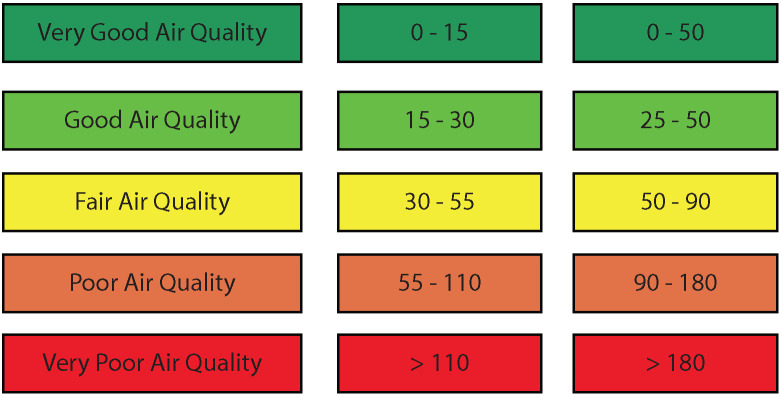
Air-quality Index (${\mathrm{PM}}_{2.5}$ and ${\mathrm{PM}}_{10}$ in $\mu {g/m}^{3}$).

Figure 5 represents the color coded visualization of pollutants’ concentrations.

#### 3.3.2. Meteorological Data

In urban areas, meteorological features are closely linked to air pollution concentrations because the generation and dispersion of air pollutants depend in part on local patterns of temperature, wind, pressure, and precipitation [35,36]. For instance, temperature affects air quality given that air near the ground is warmer than that in the troposphere. The warmer, lighter air at the surface rises and moves everything within it, including pollutants, from the ground to higher altitudes. Additionally, air pollution is easily transported from one area to another due to wind speed and wind direction. When air stops moving due to high pressure, pollutants may stagnate in one specific area, rendering this area very polluted for a period of time. Moreover, precipitations evoke what is called ’wet deposition’, which washes away most of the common air pollutants and pollen in the air when the rain is not acidic [37,38].

We therefore believe that the integration of meteorological data into our dataset is of the utmost importance. We use the API provided by OpenWeatherMap, to launch queries every 5 min using multiple zip codes and retrieve weather data. For every zip code, we fetch the temperature (°C), humidity (%), pressure (hPa), wind speed (m/s), wind direction (deg), rain (mm/1 h), and cloudiness (%).

#### 3.3.3. Traffic Data

In urban areas, traffic is one of the major sources of air pollution. General polluting emissions are produced by a wide variety of cars, motorbikes, as well as heavy duty, less environmentally friendly vehicles found in Morocco (e.g., trucks, lorries and buses). Many of these vehicles are of the aged variety, having been manufactured in an era when emission standards were different, as well as suffering from the pollutive output typically caused by aged and lower quality motors which leak far more noxious oil vapors, chemical compounds and particulate matter than a newer or more environmentally friendly counterpart would [39].

The scarcity of traffic sensors on Moroccan roads makes measuring the traffic flow a difficult task. Thus, we use a method of urban traffic data collection which consists of exploiting Google Traffic maps using image processing [40]. Traffic information is extracted from Google Traffic maps, which estimates the level of congestion on the main roads of the city of Rabat, the number of vehicles, as well as the occupancy rate on a road.

### 3.4. Geographic Data

In this section, we present the spatial features collected in our data set. To that end, we use two methods: feature abstraction and micro-level feature collection. Prior to using these two methods, we must first identify the pollution sources of concern.

#### 3.4.1. Pollution Source Identification

**Figure 6:**
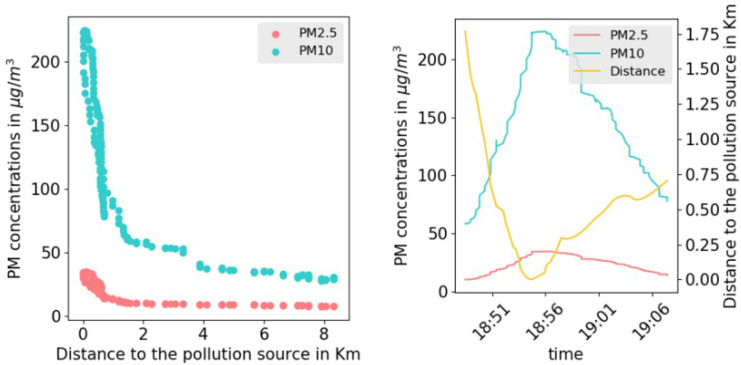
PM concentrations vs. distance to the area of interest.

In urban areas, different spatial features facilities, shops and activities are producing different amounts of pollutants, thus contributing to the rise of air pollutants’ concentrations. These sources of pollution have been identified by monitoring the levels of PM concentrations while walking in the narrow streets of the selected neighborhoods. Whenever air-quality measurements increase drastically in an area, we explored it further to identify the sources of pollution. An example of such a case was observed in a small area that hosts open-air thrift shops and street food vendors. The observed increase of the ${\mathrm{PM}}_{10}$ (resp. ${\mathrm{PM}}_{2.5}$) during the collection campaign were further validated by numerical analysis of the collected data. Figure 6 shows a strong negative correlation between the distance to the center of the area of interest (thrift shops and food vendors) and the concentration of ${\mathrm{PM}}_{10}$ (resp. ${\mathrm{PM}}_{2.5}$). The visual correlation was validated by a correlation coefficient of −0.78 (resp. −0.74) with a *p*-value in the order of $10^{-33}$ (resp. $10^{-23}$). The same observations were consistently made and tested on different days and for different sources of pollution in all the studied neighborhoods.

#### 3.4.2. Feature Abstraction

Google Maps and OpenStreetMap (OSM) are web mapping services that offer satellite imagery, street maps, and route planning. QGIS is a free and Open-Source Geographic Information System that allows people to freely access, visualize, edit and collect the global geographic data provided by these mapping services. A variety of geographic data types with detailed datasets covering many areas in the world are provided. In OSM, we can find different geographical features that include both natural and man-made elements such as: land use, roads, water areas, buildings, amenity, craft, leisure, etc. In addition, each feature can have different sub-categories. For example, the category ’buildings’ can include industrial, residential factories, and commercial structures, which could reflect the population density, traffic volumes and pollution types in a specific area. Amenity is used to map facilities used by visitors and residents, and can include banks, pharmacies, cafes, parking and schools, which can give an insight on the pollutants released to the atmosphere.

**Figure 7:**
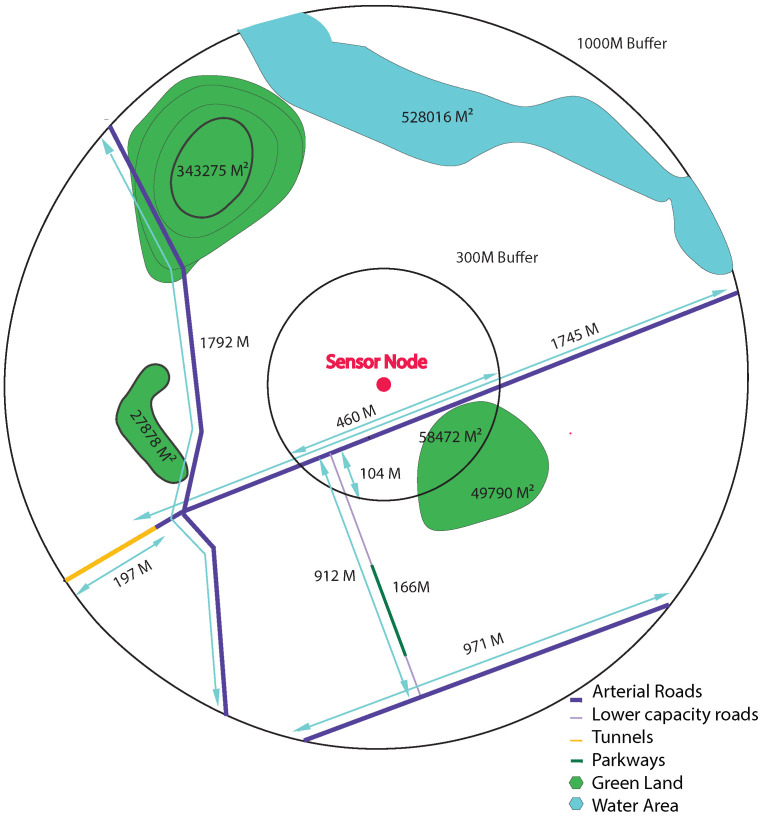
Example for available geographical features in the 500-m and 1000-m buffers using features abstraction.

To extract these data, we create, in the GIS, several buffers for each one of our sensor nodes. We consider that the monitoring station is the center of a series of circles with a radius that ranges from 100 m to 1000 m. Each circle is called a buffer. In each buffer, we collect all the geographical features available. Figure 7 is a representation of the approach we are using to extract data using GIS.

Once we have the different available features found for each buffer, we compute the sum of lengths and areas of each feature depending on its type.

For example, in the 1000 M buffer, OSM indicates the presence of three main features; green land, water areas and roads of four types: arterial roads, lower capacity roads, tunnels and parkways. It is important to specify the type of road, since it is closely related to the distribution of pollutants as mentioned in Section 2. There is a total of three green spaces, one water area and five roads of four types around the sensor node. For each line geographic feature such as the type of roads, we sum up the length of road segments within the buffer, and for each polygon geographic feature such as green land and water area, we compute the sum of overlapping areas between each type of features within the buffer. Within the 1000-m buffer, around the sensor node, we find 4508 m arterial roads, 912 m lower capacity road, 197 m tunnel and 166 m parkways as well as 454,324 $m^{2}$ green land, and 528,016 $m^{2}$ water area. Within the 300-m buffer, we find 460 m arterial roads, 104 m lower capacity road and 58,472 $m^{2}$ green land. The same procedure is repeated through all available sensor nodes to generate data for every feature type and for each buffer size. However, OSM for Morocco still lacks a lot of geographical data. Many features are not available or visible on OSM and Google street map such as building types, cuisine, public baths and ovens, and several polluting types of commerce. Thus, we have decided to take the feature collection further and collect data on a micro-level scale.

#### 3.4.3. Micro-Level Scale Data Collection

**Figure 8:**
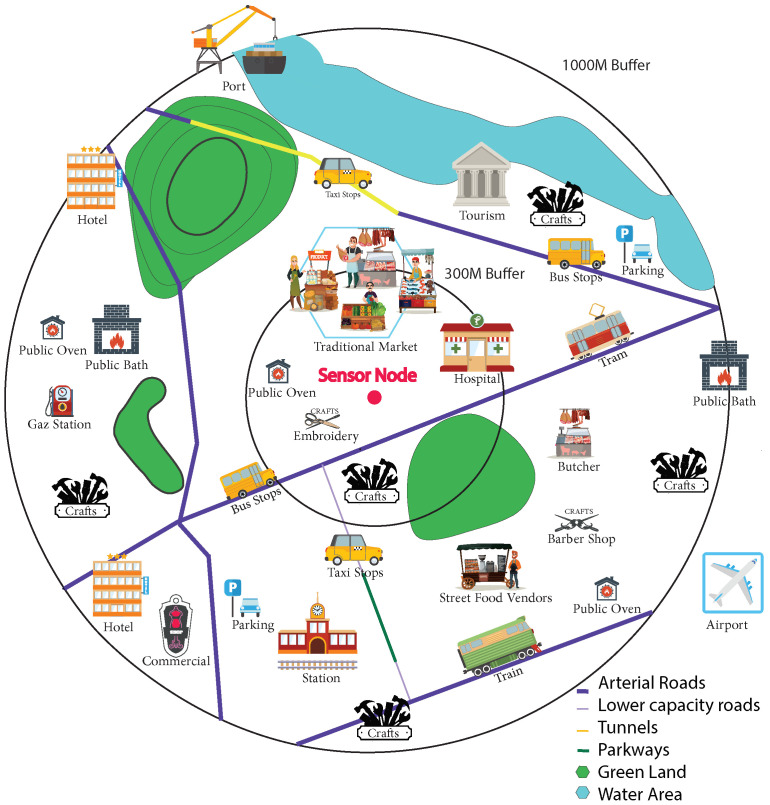
Example for available geographical features in the 500-m and 1000-m buffers.

Air pollution results from both natural and anthropogenic actions. It enters the atmosphere by different amounts and at different times. It refers to various locations, activities, and features that can either be direct sources or impacting factors for the release of pollutants. For natural sources, particulate matter (PM) come from sources such as sea salt, naturally suspended dust, pollen and volcanic ash. Man-made sources and impacting factors, however, are mostly related to the different activities found in different countries, cities, and neighborhoods. Figure 8 is an illustration of the several features that could have a potential impact on air quality and children’s health.

In some parts of Africa, many urban areas of concentrated poverty still exist and suffer risks to both human and environmental health. This is especially the case within slum settlements that face environmental challenges due to their proximity to industrial zones, city dumpsites, major highways or riparian land prone to flooding during rainy seasons, high levels of pollutants, poor air quality and other environmental hazards [41,42]. Furthermore, due to low household income, most households within slums face challenges in adopting cleaner cooking stoves and fuels, therefore propagating the reliance on dirty fuels, biomass and kerosene to meet their cooking and heating needs [43].

In our Morocco use case, we aim to find the various sources and features impacting urban air quality. As a start, we collect all potential sources and features found in the city of Rabat, Morocco’s capital. This collection is an aggregation of the existing features found in the literature and used in other countries, and the context-specific features gathered through observing the several practiced professions, activities and traditions found in Rabat, Morocco.

As with the approach used for feature abstraction, we use the buffers as a starting point and cover each buffer on foot while registering all existing features by inserting them via an application. As a start, a distance of approximately 72 km was covered on foot to collect a total of 2827 features. To take full advantage of our collection campaign, we had a pollution sensor in hand (Section 3.3), to be able to correlate and study later the impact of each feature on the pollution measurements recorded. The collection campaign was done on foot to reach areas that are very polluted but are unreachable by vehicles, such as traditional markets, narrow streets and slum areas. The different features found are explained in Table 3. Zone marker is a feature that describes building type: slum area, modern neighborhood, traditional neighborhood, modern villas and apartments, etc. Each of these types can potentially play an important role in the distribution of pollutants. Modern villas and apartments, for instance, have more green spaces and are distant from one another.

### 3.5. Dataset Summary

The rising number of disastrous effects linked to poor air quality, such as chronic diseases, global warming, and depletion of the Ozone Layer has brought to light the severity and urgency of air pollution monitoring. A first step towards air pollution management, is air pollution measurements. This paper provides a dataset of pollution measurements as well as potential sources it is coming from.

The MOREAIR dataset includes three types of environmental data: air-quality data, meteorological data and geographical data. Measurements and data are all pre-processed and provided in CSV files for each neighborhood, covering different periods of time.

This dataset, which will be available to download from the MOREAIR website (http://moreair.info/home, 2 March 2022), intends to:

(i) Alleviate the lack of open data sets related to air quality.

(ii) Enrich the literature by presenting a rigorous list of features specific to the African and Moroccan context, thus highlighting the importance of studying air pollution at regional, local and micro levels.

(iii) Provide data potentially linked to asthma and offer patients some insight to assist them in making informed decisions.

(iv) Provide open data accessible by the general public to raise awareness about air pollution in Morocco.

(v) Assist the research community by offering insight into air pollution in lower studied regions of the globe such as Africa.

## 4. Explanatory Analysis

In this section, we focus on understanding how the measured air-quality data varies in time and how the presence of various spatial features impacts their distribution in the atmosphere.

**Figure 9:**
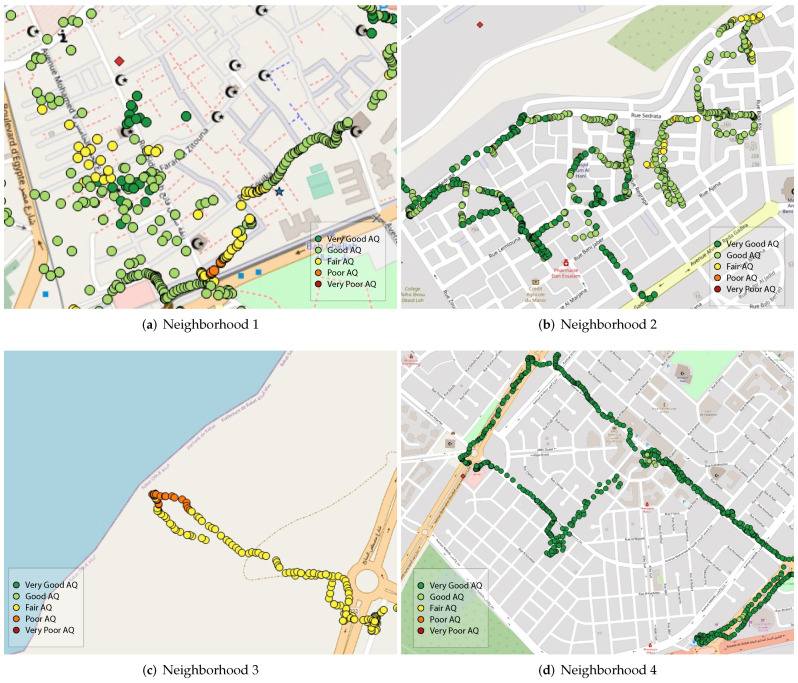
QGIS-based visualization of ${\mathrm{PM}}_{10}$ measurements collected with mobile sensor nodes.

Table 4 shows that over the period where air-quality measurements were collected, the mean concentration for ${\mathrm{PM}}_{10}$ as well as ${\mathrm{PM}}_{2.5}$ falls under 30 µg/m³ and 50 µg/m³ respectively, which are both considered to be very good air quality and represent a minimal health impact. The high particulate matter concentrations were however registered while on the move in middle-class, populous and disadvantaged neighborhoods. In Figure 9c, for instance, we can see that the closer we are to the sea, the higher the ${\mathrm{PM}}_{10}$ concentrations are, mainly due to sea salt. Figure 9a shows a representation of air quality in “El Medina”, a very populous zone with no roads or traffic, but that hosts several activities such as street food vendors, various shops, small restaurants and a few traditional markets. We can see that air quality varies from fair to poor depending on the activities found nearby. Additionally, we observe that the presence of street food vendors is linked to higher levels of air pollution compared to other activities. Figure 9d represents one of the advantaged neighborhoods in Rabat. Although it has some of the largest roads in the urban area, it still did not register any poor air quality, and most measurements showed very good air quality. We stipulate that the architecture in this area played a main role in this finding. We observed that even the larger roads in this area were often surrounded by green spaces and gardens. “Hay Nahda 1”, a middle-class neighborhood, showed good to very good air quality, except for a specific zone where many mechanical shops were found, as can be seen in Figure 9c.

When it comes to the temporal behavior of air pollution, the statistics show that the concentrations of ${\mathrm{PM}}_{10}$ and ${\mathrm{PM}}_{2.5}$ are stable during the whole week for Neighborhood 1 and Neighborhood 4, while Neighborhood 3 shows a slight peak on Friday. This can be explained by the fact that this neighborhood hosts a weekly traditional market every Friday, and the market hosts several open-air street vendors. Neighborhood 2, on the other hand, shows an increase in ${\mathrm{PM}}_{10}$ measurements on Saturday, but 31 µg/m³ is still considered to be very good air quality.

**Figure 10:**
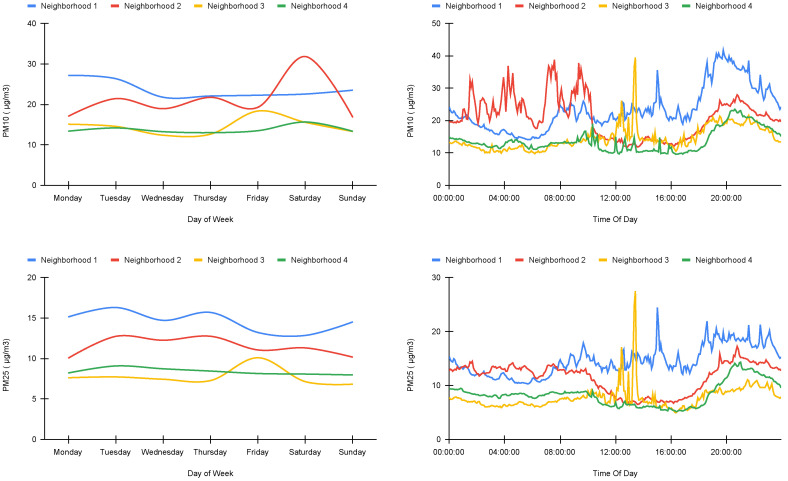
Temporal variation of PMs on four neighborhoods.

In diurnal cycle (Figure 10), the highest PM concentrations were observed during peak activity time (8 a.m., 12 a.m., 2 p.m., 6 p.m., 7 p.m., 8 p.m.) and the lowest PM concentrations were registered during trickle activity (nighttime) in most cities. The only exception was observed for Neighborhood 2, where pollution was stable and low during daytime and relatively high during nighttime. We argue that this is related to the geographical location of this site. Neighborhood 2 is located on a valley and surrounded mostly by green areas. It is known that when air mixes over a larger area, pollutants become dispersed. However, when air is stuck close to the ground, these pollutants are thicker. This low mixing height can be particularly obvious in extreme cases of “inversions”, where cold and heavy air sits on top of warmer air near the surface. Mixing height is lower at night, when air is cooler, and winds are calmer on average. This mixing height could at least partly explain why PM concentrations are higher at night and in this neighborhood.

It is worth noting that the overall registered measurements did not reach unhealthy levels in all studied sites.

## 5. Feature Importance

Feature importance is fundamental and a highly useful interpretation tool to identify and describe the features that are most relevant. From the 52 features collected, it is important to understand which ones have a higher impact on the distribution of air pollution and which ones must be included in air-quality prediction. The most common impacting factors are weather and traffic-related data.

We start from three different clusters of attributes, and select the features found inside these clusters. To decide on this matter, and effectively determine the most impacting features required for a prediction model to work properly, we use Random Forest.

Random Forest is an ensemble learning algorithm, it combines both bagging and feature randomness to create an uncorrelated forest of decision trees. Moreover, our main use for Random Forest is the built-in feature importance.

Feature importance refers to a class of techniques for assigning scores to input features to a predictive model that indicates the relative importance of each feature when making the prediction model. In our case it will help us know which features are more relevant for future use. The features importance is computed as the mean decrease in node impurity which is computed from the Random Forest structure.

After computing the feature importance for all collected features, we run the Random Forest model for both ${\mathrm{PM}}_{10}$ and ${\mathrm{PM}}_{2.5}$ separately. We included the five studied zones on the data fed to the model. Our data set includes both temporal and spatial features and since there is a wide range of temporal data and only five spatial zones, we opted to separate the two.

By including all 52 features, temporal features show a much higher impact compared to spatial ones.

**Figure 11:**
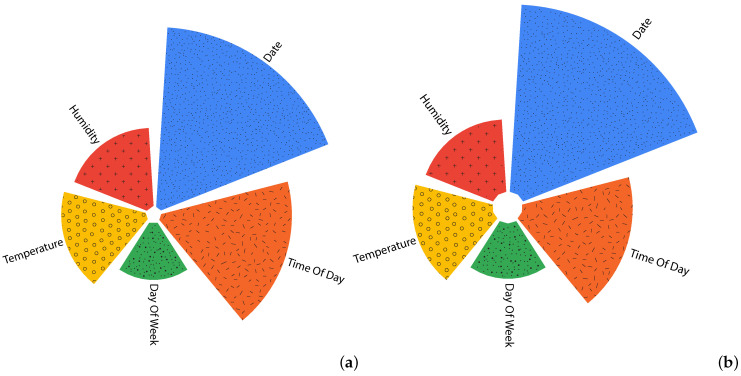
Temporal features importance for ${\mathrm{PM}}_{10}$ (**a**) and ${\mathrm{PM}}_{2.5}$ (**b**).

Figure 11 depicts the importance of temporal features, for both ${\mathrm{PM}}_{10}$ and ${\mathrm{PM}}_{2.5}$. The ’Date’ feature shows the highest importance, followed by ’Time of Day’. Meteorological factors come third where ’Temperature’ shows a higher impact compared to ’Humidity’ and day of week comes last.

**Figure 12:**
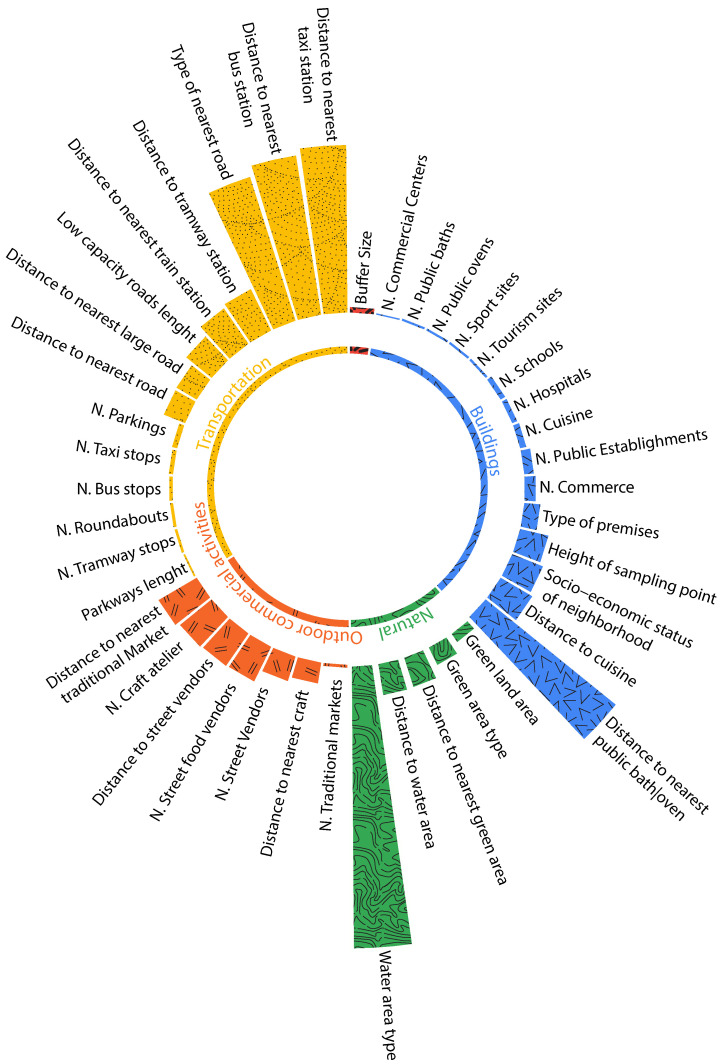
Spatial feature importance for ${\mathrm{PM}}_{10}$.

**Figure 13:**
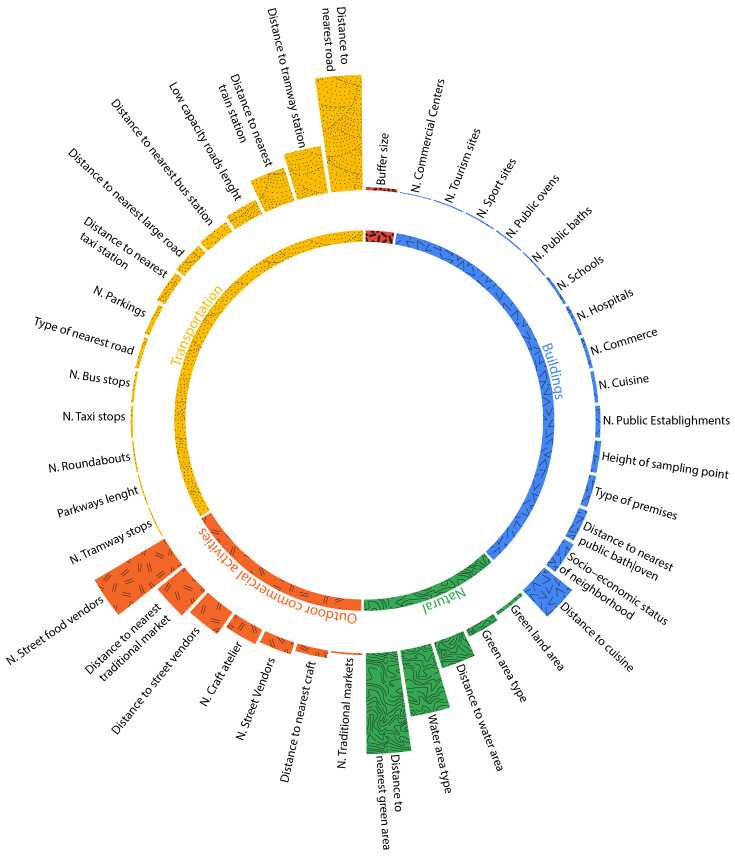
Spatial feature importance for ${\mathrm{PM}}_{2.5}$.

Contrary to temporal features, spatial features did not show the same importance for both ${\mathrm{PM}}_{10}$ (Figure 12) and ${\mathrm{PM}}_{2.5}$ (Figure 13). For a better understanding, we split the features into five categories: transportation related features, natural features, buildings related features, outdoor commercial activities, and the buffer size used while collecting the aforementioned features. Figure 12 for instance shows that for ${\mathrm{PM}}_{10}$, the most important feature is ’Water area type’. This is explained by the fact that several zones are near the beach knowing that sea salt and dust are among the most common natural sources of particulate matter. The second most important feature is ’Distance to a taxi station’ and the third is distance to public baths and ovens. As for ${\mathrm{PM}}_{2.5}$, the most important feature is ‘Distance to nearest road’, followed by ‘Distance to green area’ and by ‘number of street food vendors’.

‘Industry’, on the other hand, did not have much importance, but that is due to the fact that no industries were registered in urban areas during our data collection. Distance to pollution sources and impacting factors seemed to have a much higher impact compared to the number of these factors in specific buffer sizes.

In the ‘Buildings’ category, the main building-related source of pollution comes from public baths and public ovens, given that Morocco does not use the combustion of biomass for household heating. Public baths are indeed among the biggest users of firewood in Morocco with an average consumption of 1.5 tons per day. ‘Cuisine’ is also among the impacting factors, as mentioned before, ‘cuisine’ here means the existence of cafes, restaurants, and local food vendors. Roads and traffic-related features are among the highly impacting factors, especially taxi and bus stations, since they gather a high number of vehicles that often produce air pollution. As for outdoor commercial activities, a category that patients mainly suffer from, we find several street food vendors, which often use coal and burning wood for cooking in open air, distance to traditional markets, and number of craft ateliers that very often use the surface in front of their atelier for their work.

**Figure 14:**
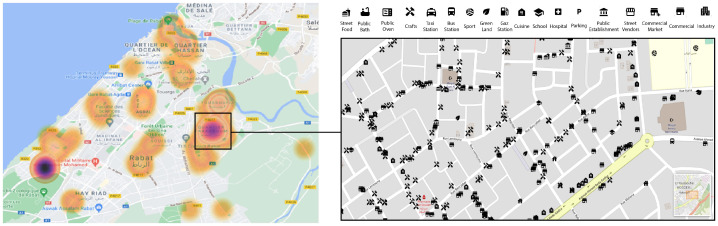
Heatmap of patient’s distribution in Rabat, and the most important features reported near their households.

Figure 14 is a heatmap visualization of the patients included in our study. Patients are distributed in different areas in Rabat, but are mostly concentrated in Neighborhood 3, Neighborhood 12, Neighborhood 10 and Neighborhood 1. These neighborhoods are the ones that showed either the highest pollution on average, or the highest variation of pollution sources registered while on the move. In Neighborhood 10, we can clearly see the sources and impacting factors collected and reported near patient’s households, which mainly include crafts, commercial activities and restaurants. Thus, small commercial shops, traditional activities, and micro-level scale features can make a great impact on the distribution of pollution and its influence on people’s respiratory state.

## 6. Conclusions and Future Work

A novel method was developed to construct the MOREAIR dataset. This method merges air-quality measurements, weather data, and all the direct or indirect impacting sources of pollution in Rabat, Morocco. In line with this objective, we start by leveraging a retrospective study of 416 children who were hospitalized for a moderate to severe respiratory disease at the Ibn Sina’s Children Hospital Center (CHUIS). The study revealed that asthma prevalence was the most common among several respiratory diseases. Patients suffering from those diseases were classified into three exposure categories (exposure to traffic, noise, and greenness). The study’s findings along with the geographical location of patients defined the placement of the deployed sensor nodes. This led to the construction of the MOREAIR dataset. Unlike its predecessors, MOREAIR contains air-quality data, as well as 52 features which were extracted by merging two methods: feature abstraction and micro-level scale data collection. Micro-Level scale data collection allowed to solve the limits encountered while using the features abstraction and to identify several context-specific sources of pollution. To identify the most important sources of pollution among the ones collected, we used the Random Forest model. This machine learning model identifies the most important features in understanding air-quality distribution in Morocco and defines the necessary factors that should be included in spatial predictions of air-quality.

By linking the medical study and the MOREAIR dataset, we observed that the patients included in our study are distributed in different areas in Rabat but are mostly concentrated in neighborhoods that showed either the highest pollution rate on average or the highest variation of pollution sources registered while on the move. The built dataset will contribute to both the environmental and medical fields and thus help patients, authorities, and the general public be aware of air quality and impacting factors present in their surroundings. MOREAIR can be extended for a larger scale and applied in different cities. The MOREAIR dataset continues to ingest new data and measurements every day from different regions of Rabat and its outskirts. We strive to expand the data collection to the rest of Morocco and to use artificial intelligence to predict air quality in time and space using the features we highlighted and the approaches we described in this paper.

## Figures and Tables

**Table 1:** Description of various respiratory diseases impacting patients in Rabat and outskirts.

Respiratory Disease	Description	Reference
Asthma	A chronic inflammatory airway disease. It is characterized by intermittent airway bronchoconstriction and mucus hypersecretion with resultant decrease in airflow and symptoms such as coughing, wheezing and breathlessness. In exacerbation of asthma, the discomfort is even more severe, oxygenation is impaired and air trapping occurs. These events are strongly linked to airway hyperresponsiveness, in which airway response to a wide variety **environmental stressors** is increased, most commonly associated with chronic exposure to **ambient air pollutants** and **tobacco smoke**. Asthma is known to be caused by; upper respiratory infections, colds, allergens such as **pollen**, **mold**, **dust mites**, as well as **tobacco**, **wood smoke**, and exposure to some pets.	[11,19,20,21,22]
Branchaleolitis	An inflammation of the bronchioles of the lungs. It is caused by viral infections and repeated exposure to animal or **vegetable dusts** usually but not exclusively. To access the lung’s tiny sacs where the oxygen is exchanged with the blood, these **dusts** must be less than a certain size, described as **5 μm**.	[23]
Acute laryngeal dyspnea	A life-threatening emergency, often caused in adults by laryngeal tumors or inflammatory edema. It can range from mild and temporary to serious and long-lasting. The most common causes of dyspnea are asthma, heart failure, myocardial ischemia, chronic obstructive pulmonary disease (COPD), pneumonia and psychogenic disorders.	[24,25]
Pneumonitis	An acute respiratory infection caused either by viral exposure or by repeated inhalation of **particles smaller in size than 5 μm**. These particles reach the lung parenchyma and evoke an immune response. Pneumonitis is not linked to any specific substances; however, many irritants could cause it, ranging from repeated exposure to some **molds** and bacteria, to exposure to feathers and birds.	[26]
Virus sequelae	A disease that occurs after a person’s body has removed a strong virus. Post-viral syndrome appears to be a reaction to the virus itself. It is often triggered by a viral infection.	[27]

**Table 2:** Information on deployed sensor nodes.

Node	Node Type	Location	Installation Date	Height of Sampling Point	Samples
ASM1	Mobile	-	-	1 m	16,873
ASN11	Nomadic	Neighborhood 1: Old Medina	7 November 2020	4 m	1,397,478
ASN12	Nomadic	Neighborhood 2: Nahda 1, Barid	11 October 2020	3 m	1,872,454
ASN13	Nomadic	Neighborhood 3: Yaacoub El Mansour	11 November 2020	6 m	2,463,693
ASN4	Nomadic	Neighborhood 4: Hay Riad	4 September 2020	3 m	2,350,808
ASN1	Nomadic	Neighborhood 5: Nahda 1 Cmpt	2 September 2020	3 m	2,680,587
ASN2	Nomadic	Neighborhood 6: Salé al Jadida	7 September 2020	6 m	2,653,698
ASN3	Nomadic	Neighborhood 7: Salé	22 September 2020	1 m	700,529
ASN5	Mobile	-	-	1 m	2200
ASN6	Nomadic	Neighborhood 8: Assabah	7 November 2020	12 m	258,526
ASN7	Nomadic	Neighborhood 9: Agdal	11 November 2020	9 m	459,577
ASN8	Nomadic	Neighborhood 10: Youssoufia	9 November 2020	1 m	106,572
ASN9	Nomadic	Neighborhood 11: Souissi	21 November 2020	3 m	668,470
ASN10	Nomadic	Neighborhood 12: EL Akkari	7 November 2020	6 m	381,380

**Table 3:** Observed features in Rabat, Morocco.

Feature Name	Sub-Features
Zone Marker	-
Gaz Station	-
Green Land	Farm - Neighborhood Garden - Valley - Abandoned land - Forest
Water Area	River - Beach - Stagnant water
Cuisine	Coffee shop - Restaurant - Bakery - Snack
Street food	Coal - No coal
Street Vendor	General - Clothes - Used Clothes - Open-Air thrift shop
Commercial	General - Small neighborhood shop - Pressing - Traveling Agency - Bank - Insurance - Library - Butcher - Fruit Seller - Clothes - Thrift Shop - Car Parts - Pharmacy - Pet Shop - Spices Shop - Drugstore - Jeweler
Commercial Center	-
Traditional Market	-
Public Bath	-
Public Oven	-
Craft	Mechanical Shop - Barber Shop - Sheet Metal Shop - Upholsterer - Carpenter - Electrician - Embroidery - Glazier - Florist - Shoe fixer - Black Smith - Carwash - Pottery - Gypsum - Zellige
Industry	-
Transportation	Tramway Stop - Bus Stop - Taxi Stop - Train Station
Tourism	Hotel - Historic - Attraction
Sport	Outdoors - Sports hall - Sports Complex
Hospital	Public Clinic - Private Clinic - Medical Office
Public Establishment	-
School	Public School - Private School - Kindergarten
Parking	-

**Table 4:** Mean ${\mathrm{PM}}_{10}$ and ${\mathrm{PM}}_{2.5}$ concentrations for each neighborhood.

Zone	Mean (${\mathrm{PM}}_{10}$)	Mean (${\mathrm{PM}}_{2.5}$)
Neighborhood 1	25.72638	15.23063
Neighborhood 2	22.059	12.56847
Neighborhood 3	18.38785	9.691565
Neighborhood 4	16.08926	9.895646
Neighborhood 5	22.29223	8.800221
Neighborhood 8	29.23791	13.62882
Neighborhood 9	29.28368	11.41109
Neighborhood 10	25.61622	14.81649
Neighborhood 11	18.77069	11.87285
Neighborhood 12	29.01119	15.4202

## Data Availability

The data presented in this study are available on request from the MoreAir website. Available at: (http://moreair.info/pollution-map, 2 March 2022).
